# A multicentric study to correlate severity of disease and vaccine in COVID-19 pandemic

**DOI:** 10.1186/s42077-022-00266-7

**Published:** 2022-10-04

**Authors:** Ankita Rushik Patel, Rekha Nilesh Solanki, Heena Sunil Channwal, Viral Rajanikant Dave, Arpit Chelabhai Prajapati, Mansi Maulik Patel

**Affiliations:** 1 Department of Anesthesiology, GCS Medical College Hospital and Research Centre, Ahmedabad, Gujarat India; 2 Department of Anesthesiology, The Gujarat Cancer and Research Institute Ahmedabad, Ahmedabad, Gujarat India; 3 Department of the Community Medicine, GCS Medical College hospital and research centre, Ahmedabad, Gujarat India

**Keywords:** COVID-19 vaccine, Severity of disease, Pandemic

## Abstract

**Background:**

The purpose of the study was to find out correlation between severity of disease and vaccine in COVID-19 pandemic. Primary objective was to know occurrence of post-vaccination breakthrough infections in hospitalized patients and secondary objective was to know of COVID-19 vaccine to prevent severe infection, morbidity, and mortality of patients. This retrospective observational multicentric study included 325 confirmed COVID-19 patients on NRBM/BIPAP/ventilator admitted in high dependency unit (HDU)/intensive care unit (ICU) were divided into based on severity of symptoms and vaccination status. We included adult patients having positive RTPCR (reverse transcription polymerase chain reaction) COVID-19 test/high-resolution computed tomography scan (HRCT) thorax suggestive of COVID-19 pneumonia. Patients who developed adverse reaction post-vaccination, pregnant patient, and lactating mother were excluded from the study.

**Results:**

Total 325 confirmed COVID-19-positive patients were studied. Statistical analysis with SPSS version 26 and data were analyzed by chi-square test and *P* value < 0.05 were considered as significant. Total duration of hospital stay was less in vaccinated patients compared to non-vaccinated patients. Oxygen requirement was also less in vaccinated patients. Vaccinated patients developed less severe infection than non-vaccinated patients.

**Conclusions:**

COVID-19 vaccination is very effective against severity of disease. It reduces hospitalization, oxygen requirement, and mortality.

## Background

We have observed 26.8 million confirmed COVID-19 cases with approximately 3.04 Lakhs deaths so far in India starting from the time of COVID-19 (Mathieu et al. 2021). In 1918, Spanish flue attacked young adults and created havoc due to secondary bacterial infection (Liang et al. 2021). In 1930, it was established that causative agent was virus but not bacteria and it took several years to get effective vaccine against it (Dahl 2018).

However, the risk of severe disease due to delta variant virus infection after vaccination remains very low (Explanation about the effectiveness of the vaccine for coronavirus 2021; Musser et al. 2021). There are some other studies also in favor of low viral load if infection occurs after vaccination as compared to unvaccinated individuals (Edara et al. 2021; Levine-Tiefenbrun et al. 2021). The purpose of this multicentric study was that to correlate severity of disease and vaccine in COVID-19 pandemic. The primary objective of the study was to know incidence of post-vaccination breakthrough infection in hospitalized patients and secondary objective was to know the efficacy of COVID-19 vaccine in prevention of severe infection and death.

## Methods

This retrospective observational multicentric study was conducted from April to July 2021 at two centers in Ahmedabad, Gujarat. The study was approved by the Institutional Ethics Committee and registered with the Clinical Trials Registry of the India (CTRI/2021/12/038722) from center 1 and center 2. After obtaining telephonic consent, mild, moderate, and severe COVID illness patients forming the study population were recruited by purposive sampling method as per their need for NRBM/BIPAP/ventilator/oxygen therapy.

The inclusion criteria were adult patients having COVID-19 RTPCR (reverse transcription polymerase chain reaction) positive/HRCT (high-resolution computed tomography scan) thorax suggestive of COVID-19 patients developing adverse reaction post-vaccination, pediatrics age group, pregnancy, and lactating mother were excluded from the study.

In COVID Opd room, we received confirmed COVID-positive either with RTPCR (by reverse transcriptase polymerase chain reaction) or HRCT thorax.

Patients had complain of the fever, headache, cold, weakness, loss of taste, and smell as per ICMR guidelines. Patients had symptoms like breathlessness, decreased oxygen saturation, increased heart rate, decreased effort tolerance, and increased work of breathing.

Patients were examined by their effort tolerance, breath holding time, 6-min walk test, and oxygen saturation, and they were admitted at designated concerned COVID areas of ward, HDU, and ICU. All the patients were segregated as per their oxygen requirement.

Detailed vaccination history including date of first dose, second dose, type of vaccine administered, any allergic reaction to vaccine were taken. History of co-morbid condition like diabetes mellitus, hypertension, chronic kidney disease, and tuberculosis was also taken.

Following parameters were observed during study:

1. Mild symptoms versus first, second, and no dose of vaccine
2. Severe symptoms versus first, second, and no dose of vaccine
3. Mode of oxygen therapy versus first, second, and no dose of vaccine
4. Length of hospital stay, discharge, and mortality versus first, second, and no dose of vaccine

## Results

The data was collected both qualitative and quantitative method and analyzed in IBPM SESS software version 20. All the table contents were calculated by using chi-square test. Inference was based on *p* value. Value of *p* < 0.05 were considered significant and *p* < 0.001 suggestive of the highly significant (Tables 1, 2, 3, and 4).

**Table 1:** Correlation of mild symptoms with first, second, and no dose of vaccine (*p* value < 0.04)

Mild symptoms versus vaccination			
Symptoms	1st dose	2nd dose	No dose
Fever	60/325	7/325	171/325
Headache	5/325	1/325	6/325
Cold	20/325	0/325	27/325
Weakness	30/325	1/325	102/325
Dry cough	108/325	5/325	177/325
Runny nose	10/325	1/325	24/325
Body ache	17/325	0/325	33/325
Loss of smell and taste	14/325	2/325	21/325

Chi-square test statistics (*p* value): 27.08 (0.040)

**Table 2:** Correlation of severe symptom with first, second, and no dose of vaccine (*p* value < 0.04)

Severe symptoms versus vaccination			
	1st dose	2nd dose	No dose
Breathlessness	37/325	1/325	114/325
Respiratory rate	19/325	1/325	97/325
Oxygen saturation	7/325	2/325	46/325

Chi-square test statistics (*p* value): 12.21 (0.049)

**Table 3:** Requirement of oxygen therapy in correlation with first, second, and no dose of vaccine (*p* value < 0.003)

Mode of oxygen therapy versus vaccination			
	1st dose	2nd dose	No dose
1 simple mask	10	5	59
2 NRBM	12	1	40
3 BIPAP	36	2	95
4 Ventilator	1	0	36

Chi-square test statistics (*p* value): 19.608 (0.003)

**Table 4:** Correlation between duration of hospital stay, discharge, and mortality of patient with first, second, and no dose of vaccine (*p* value < 0.039)

Hospital stay, discharge, and death versus vaccination			
	1st dose	2nd dose	No dose
Hospital stay			
Less than 7 days	31	4	92
7–15 days	21	4	120
More than 15 days	5	0	15
Discharge	31	4	115
Death	29	3	96

Chi-square test statistics (*p* value): 5.138 (0.039)

## Discussion

With recent developments in medical science and technology and road map created by vaccines for SARS Cov-1 and Middle East respiratory syndrome corona virus (MERS COV), we got vaccine against COVID-19 in January 2021 which is less than a year after declaration of COVID-19 as pandemic by World Health Organization (WHO) (Hindu 2021). Vaccines approved in India were COVISHIELD (Serum Institute of India), COVAXIN (Bharat Biotech/ICMR/NIV), and SPUTNIK V (Drug Controller General of India (Center for Disease Control and Prevention 2020). These vaccines have provided immunity against disease severity along with associated complications and mortality, which had threatened us mentally, physically, and economically also.

Three vaccine being used in India (COVISHIELD, COVAXIN, SPUTNIK V) have been authorized to be used only in a population of more than 18 years of age. No vaccine is superior to the other and administration must be done depending on the availability (Chauhan 2021). In our institutes, only COVISHIELD vaccine was available so that all the patients had taken this vaccine at that time.

In this retrospective study, after 1st dose of vaccine out of 325 confirmed COVID-positive patients, 108 patient had presented with dry cough, while patient who had not taking single dose of vaccine 177 where suffer from dry cough. After 1st dose of vaccine, 60 patient had present with fever, while patient who had not taking single dose of vaccine 171 where suffer from fever. After 1st dose of vaccine, 30 patient had present with weakness, while patient who had not taking single dose of vaccine 102 suffered from weakness. *P* value of this mild symptoms verses vaccine was < 0.04 suggestive of the significant.

After 1st dose of vaccine 37, 19 patient had present with severe symptoms of breathlessness and increase respiratory rate while patient who had not taking single dose of vaccine 144, 97 respectively, *P* value of severe symptoms verses vaccine was < 0.04 suggestive of significant. Any person infected with COVID-19 virus can develop a clinical course of COVID-19. However, it is reported that the most severe symptoms such as respiratory failure in older man with co-morbidities condition (Chen et al. 2020). Children and young people mostly showed a mild presentation of the disease (Xu et al. 2020).

After 1st dose of vaccine 10, 12 patient had needed simple mask, and NRBM mask while patient who had not taking single dose of vaccine 59, 40 needed respectively. Also, after 1st dose of vaccine 36, 7 patients had needed BIPAP and ventilator support while patient who had not taking a single dose of vaccine 95, 36 needed support respectively. *P* wave of the mode of oxygen therapy verses vaccine was < 0.003 suggestive of highly significant.

According to the analysis done by ICMR, of the 9.3 million who received the first dose of COVAXIN, only 4,208 tested positive; and of the 1.7 million who received the second dose, 695 tested positive. Similarly, for COVISHIELD, of the 100.3 million who received the first dose, 17,145 tested positive; and of the 15 million who got the second dose, 5014 tested positive. This translates to only around 2–4 cases per 10,000 people vaccinated (Joint Statement from the International Coalition of Medicines Regulatory Authorities and World Health Organization n.d.).

Alyson Cavanaugh et al. reported that 46 of the 199 people at the care home were infected during the outbreak at the US Center for Disease Control and Prevention. They estimated that the vaccine was effective and preventing COVID-19 infection, who had past more than 2 weeks of second dose. The dose of vaccine were even more effective at preventing hospitalization (Cavanaugh et al. 2021).

In COVID-19 vaccination, there was 95% efficacy against disease could reduce future attack rate, hospitalization, and death (Moghadas et al. 2021). It was also found that positive cases after the second dose from COVISHILD were 0.3% and 0.04% for COVAXIN, which strongly suggest that people must get vaccinated and not postponed it. These vaccines were disease-modifying vaccines. After both dose were administered, antibody develops and chances of infections decreased (Xu et al. 2020). R J Haris et al. also reported that single dose of the COVID-19 vaccine either Pfizer or Astrazenaca cuts a person’s risk of transmitting SARS-COV2 to their closest contact by as much as half. An analysis of more than 365,000 households in the UK. Although the vaccine have been shown to reduce COVID-19 symptoms and serious illness, their ability to prevent corona virus transmission has been unclear (Haris et al. 2021).

M. G. Thompson et al. noticed that full immunization was 90% effective protecting people against infection and a single dose was 80% effective but researchers caution that because every few participant became infected after vaccination, it is difficult to state the vaccines effectiveness against infection with high precision (Thompson et al. 2021).

Since there was no guarantee of any vaccine being 100% effective, we expect COVID infection in some individuals even after full vaccination. In many countries, there was higher rate of breakthrough infection with delta dominant variant.

## Conclusions

This study showed that COVID-19 vaccination is very effective against severity of disease. It is thus imperative for healthcare workers to educate, spread awareness, quote authentic data, motivate, communicate, and bring clarity in the minds of all who are doubting this process of vaccination. Along with this main preventing measure of S-sanitation, M-mask wearing, and S-social distancing is the only way forward to save us from the catastrophe of corona virus infection.

## Data Availability

The dataset during the current study are available from the corresponding author on reasonable request.

## Abbreviations

SARS: Severe acute respiratory syndrome
CRP: C-reactive protein
LDH: Lactate dehydrogenase
ABGA: Arterial blood gas analysis
NRBM: Non-rebreathing mask
HDU: High dependency unit
ICU: Intensive care unit
RTPCR: Reverse transcription polymerase chain reaction
HRCT: High-resolution computed tomography
